# Clinical effectiveness of high definition fluorescence camera in detection of initial occlusal caries

**DOI:** 10.4317/jced.59185

**Published:** 2022-02-01

**Authors:** Mohamed Salama, Olfat Hassanein, Omar Shaalan, Asmaa Yassen

**Affiliations:** 1 Doctoral Candidate, Department of Conservative Dentistry, Faculty of Dentistry, Cairo University. Assistant Lecturer, Department of Conservative and Esthetic Dentistry, Faculty of Dentistry, The British University in Egypt; 2 Professor, Department of Conservative Dentistry, Faculty of Dentistry, Cairo University; 3 Lecturer, Department of Conservative Dentistry, Faculty of Dentistry ,Cairo University; 4 Professor, Department of Conservative Dentistry, Faculty of Dentistry, Cairo University; Department of Conservative and Esthetic Dentistry, Faculty of Dentistry , The British University in Egypt

## Abstract

**Background:**

Although visual inspection is the preferred route in everyday clinical practise for detecting early caries lesions, novel technologies like light fluorescence-based devices (Vista Proof iX HD smart) have been developed to enhance early caries detection.

**Material and Methods:**

Occlusal surface of 45 molar and 49 premolar teeth from 34 adult participants who fulfilled the eligibility criteria were examined by two observers using three diagnostic methods. Examination was performed visually using the International Caries Detection and Assessment System (ICDAS-II) followed by Vista Proof. Fissurotomy was applied for histological validation. Intra- and inter-observer agreement were measured for ICDAS-II and light-induced fluorescence camera using Kappa test. The overall diagnostic accuracy parameters, area under the receiver operating characteristic curve (AUC-ROC) and 95% confidence interval (95% CI) of the (AUC) for caries detection by Vista Poof were evaluated.

**Results:**

For ICDAS-II and Vista Proof methods, there was almost perfect intra- and inter-observer agreement. Based on ICDAS-II as a reference standard 1, Vista Proof showed a low level of agreement in enamel carious lesion detection with low sensitivity value of 48%, high specificity of 100%, and AUC was 0.112, while a high level of agreement in dentin carious lesion detection with high sensitivity value of 100%, low specificity of 48% and AUC was 0.888. Based on fissurotomy as reference standard 2, Vista Proof showed a high level of agreement in dentin carious lesion detection with high sensitivity value of 95% and 0% specificity and AUC was 0.814.

**Conclusions:**

Quantitative light-induced fluorescence camera with reference to ICDAS-II is considered as an accurate diagnostic modality for detection of early occlusal caries. Histological findings validate the diagnostic accuracy of the camera in dentin.

** Key words:**Histological validation, Initial caries, ICDAS, Light induced fluorescence, Vista Cam.

## Introduction

First and foremost, dental caries is a disease that affects 60-90 % of the total of school-aged children and adults worldwide (1). To adequately manage this globally prevalent condition, it appears that a thorough understanding of dental caries and its associated variables is required (2). The management of dental caries has changed as our understanding of disease has evolved. Diagnosis of occlusal caries in initial stage is considered problematic and challenging for dental professors, due to complicated anatomy of groove-fossa system, presence of staining, and deposition of plaque, calculus, or other substances that might interfere with accurate diagnosis (3).

Clinically, caries diagnosis is commonly performed by multiple methods as visual tactile methods, assessments of translucency, color, and dental hardness, as well as by means of radiographic imaging. International caries detection and assessment system (ICDAS) based on standard method for visual diagnosis of dental caries had been widely used as gold standard for clinical diagnosis of dental caries (4). The visual inspection has various limits in its application. The most evident is that it is relied on practitioner’s subjective evaluations, thus lesions can go undiagnosed because teeth are often viewed with naked eye (5).

As a result, occlusal caries detection technologies should not only be capable of detecting and monitoring lesions at all phases of caries process, but also be very reliable. Because detecting early occlusal caries is challenging, novel approaches for detecting early caries have been developed. Some of these technologies use light-based fluorescence devices to utilize fluorescence properties of hard tissues (6). The light-induced fluorescence of bacterial by-products or tooth structure is used to diagnose diseased teeth. Limited data about clinical performance of a high-definition quantitative light fluorescence-based camera in detection of initial caries are available. So, this investigation was carried out to validate its accuracy. The null hypothesis tested is that there is no difference in the reliability of quantitative light fluorescence-based camera (Vista Proof) in comparison with ICDAS-II and fissurotomy validation in detection of initial occlusal caries.

## Material and Methods

The current study’s protocol was registered in protocol registration and results system (www.clinicaltrials.gov) database under identification number NCT03940170. All procedures involving human subjects in this study were carried out in accordance with ethical requirements of Research Ethics Committee of Faculty of Dentistry, Cairo University (Approval number. CREC 19628). The outpatient clinic of Conservative Dentistry department, Faculty of Dentistry, Cairo, Egypt, hosted this diagnostic clinical trial and each participant that included in this study signed an informed consent form after describing research procedure in-depth.

Based on previous study by Presoto *et al*. 2017 (7) in which Area under ROC curve for diagnostic accuracy of fluorescent camera was 0.777 and 0.914 for ICDAS-II, it was estimated that both methods would need a minimum of 94 teeth. Calculation was performed using MedCalc 12.4.0 software.

Participants were enrolled according to the following criteria: All patients were at least 18 years old having at least one suspected posterior pits and fissure with occlusal discoloration. Exclusion criteria included teeth with fluorosis, hypoplasia, amelogenesis imperfecta, hypomineralization, pit and fissure sealants/restorations, and third molars. (7,8). According to these eligibility criteria, 34 participants were enrolled: 15 females and 19 males. Ninety-four teeth were included in the study: 45 maxillary and 49 mandibular teeth. Premolars comprised 52.1% and molars comprised 47.9% of the examined teeth.

**Figure 1:**
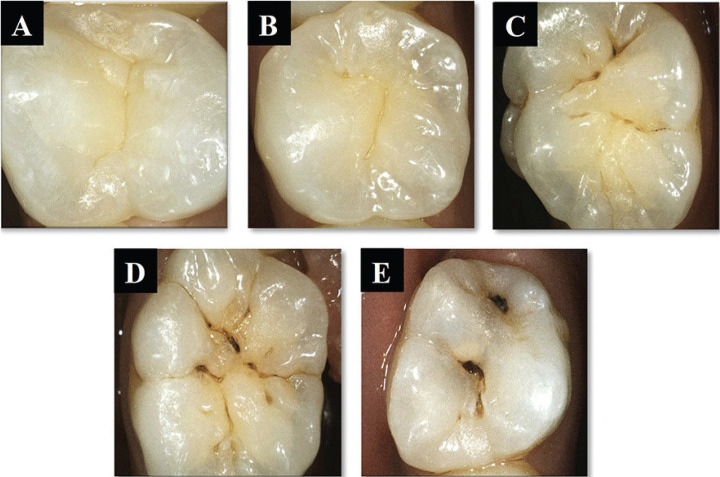
A-E: ICDAS-II scoring system representing score from 0 – 4 with intraoral images: (A) Code 0; (B) Code 1; (C) Code 2; (D) Code3; (E) Code 4.

Each lesion was evaluated using ICDAS-II, a light-induced fluorescence camera, and fissurotomy as a confirmatory assessment. Fifteen days before the inquiry, the two examiners (MS and AY) assessed 60 extracted teeth for calibration. Each examiner diagnosed the teeth and recorded their results. They compared the results and returned the discrepancy cases until they reached 100% consistency (7). True blinding was not applicable, otherwise, obtained data from each examiner was not exchanged with the other examiner. Each examiner conducted diagnostic procedures in a separate cabin. Visual examination was conducted first before assessment using light-induced fluorescence method. This minimized the risk of assessment bias. Furthermore, a sequential clinical examination for all enrolled participants was conducted as follows: In first visit, teeth were carefully scaled and polished to remove surface biofilm and calculus deposits using ultra sonic scaler. Furthermore, teeth were cleaned for 10 seconds with a water powder jet cleaner (Prophy Neo Mate, NSK, Japan) using calcium carbonate spherical powder (Flash pearl polishing powder, NSK, Japan), followed by another 10 seconds rinsing with an air water spray for thorough cleaning of fissures from any powder residues. Teeth were examined by two examiners using two diagnostic modalities under standardized conditions of lightening from dental unit and by using front surface mirror (Zeffiro, Italy), oil free air syringe and CPITN probe (HAHNENKRATT, Germany). Teeth were examined visually while they were wet then after being dry for 5 seconds with a triplex syringe. Examiner noted changes in tooth translucency, opacity, or color, compared to adjacent healthy teeth, and classified their ICDAS scores (9) (Fig. 1). Magnification was not used with visual examination as recommended by ICDAS modality. Highest ICDAS score in each occlusal surface was recorded at investigation site.

**Figure 2:**
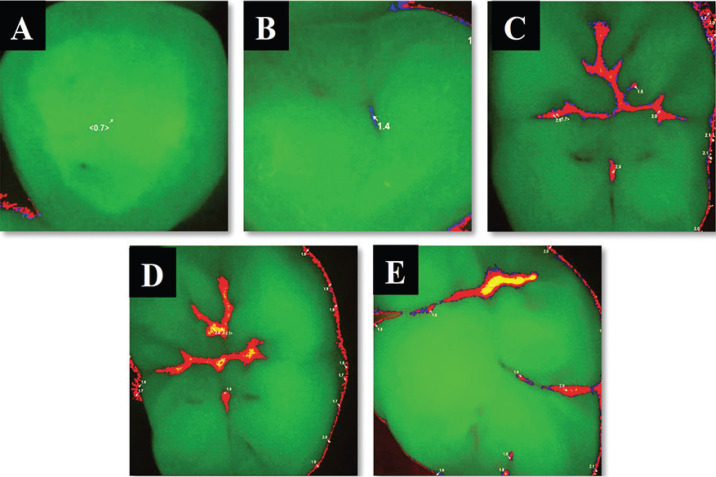
A-E: Vista Cam scoring system according to manufacturer with intraoral representative fluorescence images: (A) score 0≤ x<1 ;(B) score1≤x<1.5; (C) score1.5≤x<2; (D) score2≤x<2.5; (E) score x ≥2.5.

Regarding quantitative analysis of lesion activity using Vista Proof Camera, teeth were examined using fluorescence-induced interchangeable head of the camera (Vista Proof) following manufacturer’s instructions. The head of the camera was positioned perpendicular to the occlusal surface of teeth. This scenario was conducted under isolation with cotton rolls and suction tip and after tooth drying with a triplex air syringe for 15 seconds. By pressing focus button, the camera focused sharply on the tooth followed by pressing the trigger button for capturing the image. Image was analyzed by special software (DBSWIN) version 5.15.1. The software produced a digital image that showed lesions in different colors with numerical values between 0 and 3 predicting depth and extent of the lesion Figure (2) (10).

The true histological extent of carious lesions was determined by fissurotomy which was considered as a confirmatory test. Clinical fissure evaluation was decided according to the reading obtained from Vista Cam, ICDAS-II, in addition to lesion activity and caries risk assessment. When Vista Cam reading ≥ 2 and ICDAS-II as score 3, fissurotomy was carried out. In case of ICDAS-II score 2, fissurotomy was only carried out when Vista Cam reading was ≥2, and the lesion appeared to be active on visual (matt appearance) and tactile (soft) examination and the participant was categorized as high caries risk (11). It is worth mentioning that current literature regarding ICDAS-II score 2 reported that dentin involvement was evident upon histological validation in several teeth within this score (12). The procedure was done using fissurotomy kit (SS WHITE USA). Suitable sixed bur gently went over the fissure and then it was visually inspected under magnification loupes (4x custom made Univet loupes, Italy). Extent of the lesion was examined with the tip of exploratory probe to assess hardness of the bottom of the fissure. Final depth of the lesion was taken to represent the ‘true lesion extent’ (code1: enamel and code2: dentin).For subsequent evaluation of both examiner’s scores, ICDASII scores, corresponding Vista Proof reading and fissurotomy were represented in  Table 1. Management of dental condition was done for patient’s satisfaction and health care (13).

**Table 1:**
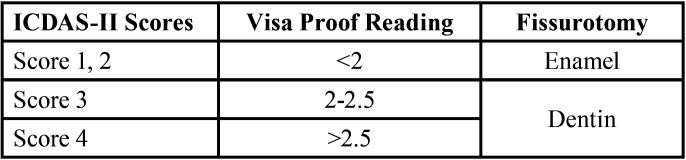
Description of visual, fluorescence-based diagnostic criteria and fissurotomy.

Statistical analysis was performed using IBM SPSS Statistics Version 20 for Windows. Significance level was set at *P* ≤ 0.05. Intra- and inter-observer agreement regarding ICDAS-II and Vista Proof modalities were evaluated using Cohen’s Kappa test. Diagnostic validity of Vista Proof device was determined, including sensitivity, specificity, overall accuracy, positive and negative predictive values, and ROC curve analysis, in detection of caries compared with traditional ICDAS-II and detection of enamel and dentin lesions compared with fissurotomy and ICDAS-II.

## Results

**Figure 3:**
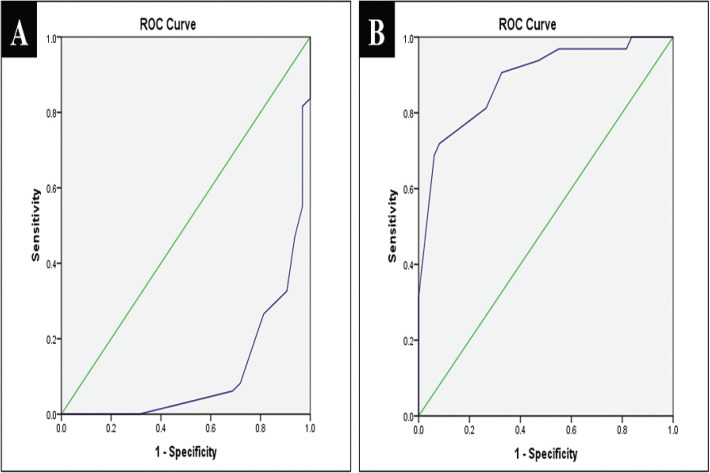
A,B: ROC curve between Vista Proof and ICDAS-II (A)enamel carious lesions; (B) dentin carious lesions.

**Figure 4:**
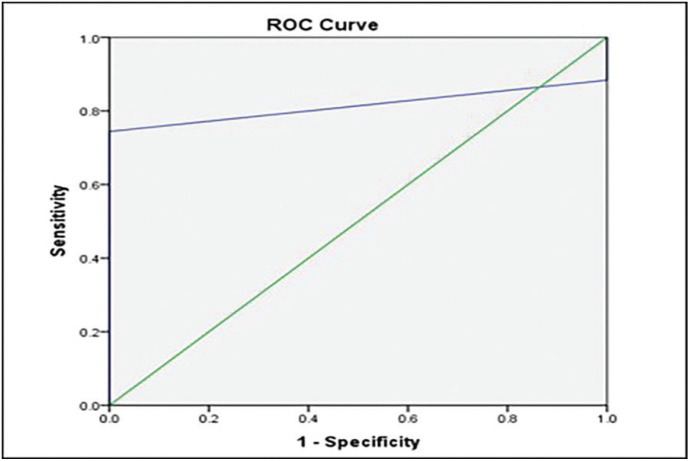
ROC curve between Vista Proof and fissurotomy in detection of dentin carious lesions.

There was nearly perfect intra-observer agreement for ICDAS-II (Kappa= 0.943 for observer 1 and Kappa= 0.891 for observer 2), and Vista Proof methods (Kappa = 0.841 for observer 1 and Kappa= 0.810 for observer 2). Inter-observer agreement was nearly perfect for both ICDAS-II and Vista Proof methods (Kappa= 0.854 and Kappa= 0.872, respectively). Vista Proof showed a low level of agreement with ICDAS-II in enamel carious lesion detection with low sensitivity value of 48%, high specificity of 100% to achieve an overall accuracy of 67% (Table 2). Positive and negative predictive values were 100% and 53% respectively showing a slightly better predictive value for carious teeth than sound teeth. ROC curve analysis revealed that AUC was 0.112 with 95% confidence interval (0.037-0.187), which indicates a poor association between ICDAS-II and Vista Proof methods. While Vista Proof showed a high level of agreement with ICDAS-II in dentin carious lesion detection with high sensitivity value of 100%, low specificity of 48% to achieve an overall accuracy of 67%. Positive and negative predictive values were 53% and 100% respectively showing a slightly better predictive value for sound teeth than carious teeth. ROC curve analysis revealed that AUC was 0.888 with 95% confidence interval (0.813-0.963), which indicates a good association between ICDAS-II and Vista Proof methods (Table 2, Fig. 3). Vista Proof showed a high level of agreement with fissurotomy in dentin carious lesion detection with high sensitivity value of 95% and 0% specificity to achieve an overall accuracy of 95% (Table 3). Positive and negative predictive values were 95% and 0% respectively showing a slightly better predictive value for carious teeth than sound teeth. ROC curve analysis revealed that AUC was 0.814 with 95% confidence interval (0.689-0.939), which indicates a good association between fissurotomy and Vista Proof method (Table 3, Fig. 4).

**Table 2:**
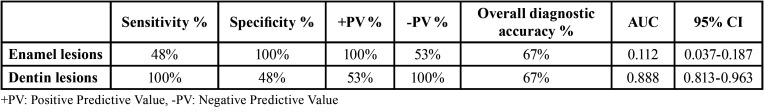
Diagnostic accuracy of Vista Proof method in detection of enamel and dentin carious lesions based on ICDAS-II as reference standard.

**Table 3:**
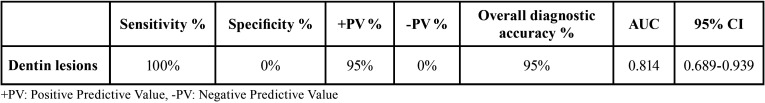
Diagnostic accuracy of Vista Proof method in detection of dentin carious lesions based on fissurotomy as reference standard.

## Discussion

Early detection of caries is the corner stone in deciding whether to perform preventive rather than surgical treatment strategies. Delay in caries treatment may occur if caries is not recognized or underestimated, thus resulting in deep carious lesions extending to dentin. In this study, international caries detection and assessment system (ICDAS-II) was taken as reference standard for detection of diagnostic accuracy of fluorescence camera (Vista Proof iX HD Smart) in addition to use of fissurotomy technique not histopathological cross section for a histological validation of results. The main challenge for using histopathology as reference standard is that it is limited to the *ex-vivo* studies or can only be performed in clinical studies with a relatively small number of teeth that can be extracted and having laboratory examination (14). In addition, it has been recently shown that stereomicroscopic examination for histopathological examination had low accuracy in detecting dentin demineralization and underestimated the real depth of dentin involvement (15). Kappa values for intra- and inter-observer agreement level in current visual examination using ICDAS-II system showed that there was almost perfect agreement between measurements. This outcome may be related to the examiners’ prior training in utilizing the ICDAS-II scoring system. Inter-observer agreement results agreed with Jablonski-Momeni *et al*. (2008) (16). While Rodrigues *et al*. (2008) (17) discovered that inter-examiner values for ICDAS-II scoring system were slightly lower than current findings. Such results could be attributed to various clinical experiences among examiners and short calibration periods prior to testing.

The results of intra-observer agreement and inter-observer agreement for Vista Proof iX HD Smart as presented by kappa values showed that there was almost perfect agreement between measurements. This could be attributed to examiner’s training on how to use the new diagnostic fluorescence camera, as well as taking a reading of exact examination site on occlusal surface for each tooth every time the tooth was examined. In addition to using the spacer, which allowed the image position and distance to be reproduced and reduced the penetration of external light. Inter-observer agreement results agreed with Jablonski-Momeni *et al*. (2012) (18). This finding disagreed with the findings of Novaes *et al*. (2012) (19), who discovered that inter-examiner values for Vista Proof iX were slightly lower than current findings. This could be attributed to examiners’ lack of experience with the device, as well as an issue with acquiring focus during image capture by the device, which was solved in the latest released version of the device (Vista Proof iX HD Smart) by integrating autofocus bottom. In addition to difference in methodology between studies, especially between vivo and vitro studies, the effect of storage media such as chloramine, formalin, or thymol solution *in vitro* studies resulted in a decrease in porphyrin-based fluorescence (20).

Florescence camera (Vista proof HD smart) showed a low level of agreement with ICDAS-II in enamel carious lesion detection with low sensitivity high specificity, an overall accuracy of 67% with a very poor AUC .These results could be justified by the fact that the device is very sensitive to any changes in the carious lesion with great sensitivity to any bacterial biproducts which made its scores exceeding the scores of ICDAS II. On the other hand, other studies revealed the inability of the device to quantify the scattered fluorescence light in the early demineralized enamel areas as it could not measure the intrinsic changes in enamel structure, so it has a lower performance for early enamel lesions. In addition, the type of enamel affects the ways of scattering of the device (21). Moreover, porphyrins, which are a product of bacterial metabolism was found to be less on the enamel surface(22). This was in agreement with (12,19,21) which mentioned that Vista Proof might have certain difficulties in detecting caries at this level showing low sensitivity. While this finding disagreed with the findings of (8,23,24) and which reported that VistaCam iX was characterized by a high sensitivity and low specificity. This might be referred to the changes in the cutoff points that were used in the methodology of these studies in addition to using an older version of the device. These cutoffs should be interpreted with caution, as a 0.1 difference can cause the score to shift from sound to carious enamel or from carious enamel to dentin (17,25).

Florescence camera (Vista Proof HD smart) showed a high level of agreement with ICDAS-II in dentin carious lesion detection with high sensitivity, low specificity , an overall accuracy of 67% and a very good AUC. This high sensitivity can be explained as the red fluorescence monitored by the device as caries, was related to the microbial metabolic products which were expected to be present in larger amounts in such dentin lesions (21) . Also, higher Fluorescence occurred in dentin due to the higher concentration of organic molecules was brighter than that of enamel (26). This finding was in agreement with (17,27) that obtained high sensitivity at the dentin level. Low specificity might be referred to the effect of stain inclusion especially the dark satins in the lesions and improper cleaning of the fissures resulting in higher measurement values and consequently in higher rates of false positives (28). This finding was in disagreement with (24) that showed low sensitivity and high specificity in dentin lesion that might be related to that most of the scores were in deep dentin and using of storage media which lead to the decrease of porphyrin-based fluorescence (20), in addition to it was claimed that the carious area of the highest ICDAS score might appear dark due to implied extensive scattering and absorption phenomena. This could explain the weak fluorescence intensity on the infected area which can underestimate the light induced camera reading in deep dentin carious 

The level of agreement between Vista Proof HD smart and fissurotomy in detection of dentin lesions showed strong correlation between the two modalities. However, in this study validation of enamel lesions efficiently could not be achieved due to ethical issues. Where ICDAS II scores 0 and 1 were assessed with no further validation by fissurotomy, as they were indicated for remineralization and not pits and fissures sealing. ICDAS-II score 2 was validated depending on individual caries risk assessment, lesion activity and when Vista Proof HD smart score was ≥ 2. Follow-up appointments for re-assessment of teeth with ICDAS-II score 0-2 was conducted to monitor lesions progression.

Upon histological validation, the camera showed a high sensitivity and 0% specificity, an overall accuracy of 91% with a very good AUC. These results have confirmed the preceded outcome which stated that the Vista Proof had high sensitivity in detection of dentin lesion. The poor specificity can be related to the absence true negative reading (Only carious lesions were opened), which led to 0% specificity and 100% sensitivity. This finding was in accordance with Melo *et al*. (2015) and Melo *et al*. (2017) (8,29), while it was in discordance with Diniz *et al*. (2012) and Jablonski-Momeni *et al*. (2014) (12,30). This conflict might be due to difference in methodology such as including exclusion and inclusion criteria of the teeth or differences in cut-off points among different studies, and storage times and media used in case of *in-vitro* or *ex-vivo* studies.

Following the analysis of study results, the null hypothesis of this study was partially accepted as light-induced fluorescence camera was equivalent to ICDAS-II modality in distinguishing carious and non-carious teeth. However, it exceeded ICDAS-II in distinguishing between enamel and dentin caries. Histological validation agreed with Vista Cam scores in dentin carious lesions.

## Conclusions

Under the conditions of the present study, the following conclusions might be drawn:

1- Quantitative analysis of carious lesions is crucial in achieving preventive dentistry rather than using a subjective visual assessment.

2- Quantitative light-induced fluorescence camera with reference to ICDAS-II is considered as an accurate diagnostic modality for detection of early occlusal caries.

3- Histological findings validate the diagnostic accuracy of the camera in dentin carious lesions.
